# Development of a physiologically based pharmacokinetic (PBPK) population model for Chinese elderly subjects

**DOI:** 10.1111/bcp.14609

**Published:** 2021-05-18

**Authors:** Cheng Cui, Jie En Valerie Sia, Siqi Tu, Xiaobei Li, Zhongqi Dong, Zhiheng Yu, Xueting Yao, Oliver Hatley, Haiyan Li, Dongyang Liu

**Affiliations:** ^1^ Drug Clinical Trial Center Peking University Third Hospital Beijing 100191 China; ^2^ School of Pharmaceutical Sciences Tsinghua University Beijing 100084 China; ^3^ School of Pharmaceutical Sciences Peking University Health Science Center, Peking University Beijing 100191 China; ^4^ Janssen China R&D Center Shanghai 200233 China; ^5^ Certara UK Ltd, Simcyp Division Sheffield S1 2BJ UK; ^6^ Department of Cardiology and Institute of Vascular Medicine Peking University Third Hospital Beijing 100191 China

**Keywords:** Chinese Geriatric Population, CYP1A2, CYP3A4, PBPK, Renal Excretion

## Abstract

**Aims:**

This study aims to develop and verify a physiologically based pharmacokinetic (PBPK) population model for the Chinese geriatric population in Simcyp.

**Methods:**

Firstly, physiological information for the Chinese geriatric population was collected and later employed to develop the Chinese geriatric population model by recalibration of corresponding physiological parameters in the Chinese adult population model available in Simcyp (i.e., Chinese healthy volunteer model). Secondly, drug‐dependent parameters were collected for six drugs with different elimination pathways (i.e., metabolized by CYP1A2, CYP3A4 or renal excretion). The drug models were then developed and verified by clinical data from Chinese adults, Caucasian adults and Caucasian elderly subjects to ensure that drug‐dependent parameters are correctly inputted. Finally, the tested drug models in combination with the newly developed Chinese geriatric population model were applied to simulate drug concentration in Chinese elderly subjects. The predicted results were then compared with the observations to evaluate model prediction performance.

**Results:**

Ninety‐eight per cent of predicted AUC, 95% of predicted *C*_max_, and 100% of predicted CL values were within two‐fold of the observed values, indicating all drug models were properly developed. The drug models, combined with the newly developed population model, were then used to predict pharmacokinetics in Chinese elderly subjects aged 60–93. The predicted AUC, *C*_max_, and CL values were all within two‐fold of the observed values.

**Conclusion:**

The population model for the Chinese elderly subjects appears to adequately predict the concentration of the drug that was metabolized by CYP1A2, CYP3A4 or eliminated by renal clearance.

**What is already known about this subject**

- Several physiologically based pharmacokinetic (PBPK) models have been developed to predict drug exposure in Caucasian elderly subjects, while little application of the PBPK model has been made in Chinese elderly subjects due to lack of a Chinese geriatric population model.
- The Caucasian geriatric population model or the Chinese adult population model may not be applicable to the Chinese geriatric population due to different PK observed in these populations, suggesting different physiology in the Chinese geriatric population.

**What this study adds**

- A population model for the Chinese geriatric population at the age of 65 and above, including subjects that are older than 75, was developed for the first time.
- This population model improved the PBPK model prediction performance in Chinese elderly subjects when compared with the Caucasian geriatric and Chinese adult population models.
- The PBPK model with this newly developed population model can reasonably predict the pharmacokinetics of drugs metabolized by CYP1A2, CYP3A4 or eliminated by renal clearance in Chinese elderly subjects with less than two‐fold error.

## 1 INTRODUCTION

Elderly subjects aged over 65 are the largest population in the pharmaceutical market that takes an average of two to five kinds of medication per day.^1^ However, it is also one of the least studied populations during drug development as they are generally not included in the clinical trial due to complex pathophysiology, variability in organ function and presence of co‐medication.^2^ Thus, the approved dosing regimen for young adults has usually been applied to geriatric patients, which may not be appropriate since the drug absorption, distribution, metabolism and elimination (ADME) in geriatric patients may be different.^3^

In the absence of well‐designed clinical trials focusing on geriatric patients, modelling and simulation technique may serve as an alternative way to understand pharmacokinetic (PK) and pharmacodynamic (PD) variability and to inform dosing regimen design. Physiologically based pharmacokinetic (PBPK) modelling can describe the drug ADME process by incorporating drug properties and physiological variables from a specific population. Over the past several years, it has been successfully applied to predict drug concentration in paediatrics, pregnant women and renal impairment patients.^4^, ^5^, ^6^, ^7^, ^8^

To date, a few studies have been conducted on PBPK models to predict drug concentration in the geriatric population. In these models, alterations of certain physiological parameters with ageing were considered, such as alveolar ventilation, cardiac output, different organ (e.g., liver, brain, heart, kidney, spleen, left and right lung) weights and blood flows.^9^, ^10^, ^11^, ^12^, ^13^ However, most models were developed in the Caucasian geriatric population whose physiological variables and PK characteristics are not necessarily the same in the Chinese geriatric population. In fact, such difference has been observed between Caucasian and Chinese young adults. For instance, the differences in the frequency of cytochrome P450 (CYP)2C19 poor metabolizers (PMs), CYP2D6 PMs, and intermediate metabolizers (IMs) caused a reduction in clearance of phenacetin, omeprazole, desipramine, midazolam and alprazolam by 19–75% in Chinese subjects, compared to Caucasian subjects.^14^ Thus, it may be risky to predict drug exposure in the Chinese geriatric population by adopting the Caucasian population model directly. On the other hand, elderly subjects usually have different PK characteristics from young people due to the degeneration of organ function, which also hampers the application of the Chinese adult population model to predict PK characteristics of the Chinese elderly subjects. For example, the clearance of theophylline after oral administration in people aged 62–93 was approximately 54% lower than in people aged 21–24.^15^, ^16^ Thus, it is necessary to develop a population model that is specific to the Chinese geriatric population.

Li et al. previously developed a Chinese population model by incorporating demographic information, microsomal protein per gram of liver (MPPGL), liver weight and CYP1A2 abundance that were specific to the Chinese population.^17^ This model was used to predict theophylline concentration in Chinese geriatric subjects and achieved reasonable prediction with predicted maximum drug concentration (*C*_max_) and area under the curve (AUC) within two‐fold of the observed values. However, most of the demographic information came from Chinese young adults and thus limited the application of this model to much older Chinese subjects. Besides, this model did not account for the change of cardiac output and liver blood flow in the geriatric population. These may have little impact on accurate prediction of PK of drugs with low hepatic extraction ratio (such as theophylline in the study) but are important physiological parameters for predicting PK of medications with a medium or high hepatic extraction ratio.

The objectives of our study are (i) to develop a PBPK population model for Chinese elderly subjects aged 65 and above, notably including those older than 75, based on more comprehensive physiological data; and (ii) to verify the Chinese geriatric population model using six substrates specifically eliminated by different pathways.

## 2 METHODS

**FIGURE 1:**
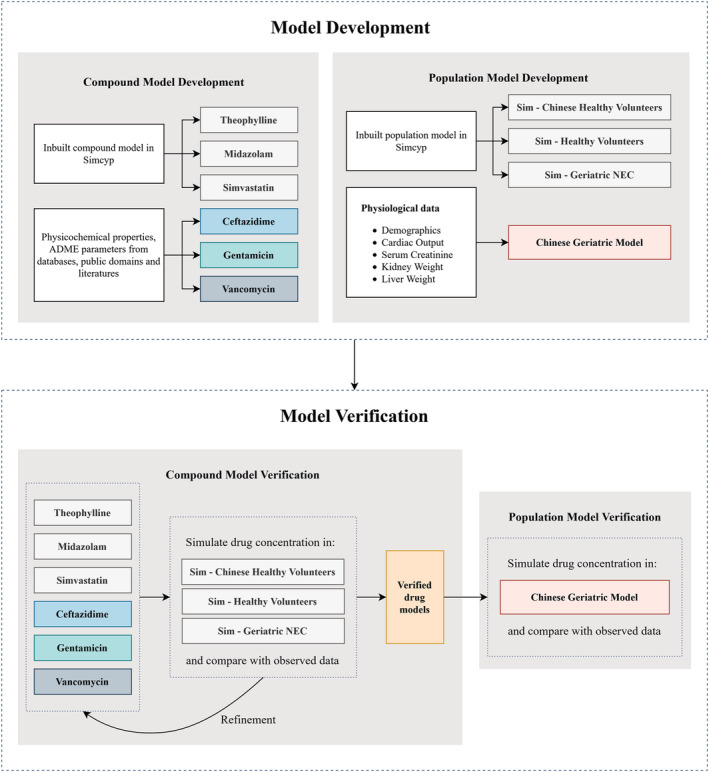
Overall workflow of the Chinese geriatric physiologically based pharmacokinetic (PBPK) population model development and verification

Figure 1 illustrates the overall workflow for the Chinese geriatric population model development and verification in Simcyp (18R1; Certara UK Ltd, Simcyp Division, UK). Briefly, physiological information from the Chinese elderly subjects, including demographic details, cardiac output, liver weight, kidney weight and serum creatinine was obtained from the public domain. The collected data was then employed to develop the Chinese geriatric population model by recalibration of these physiological parameters in the Chinese adult population model available in Simcyp.^18^ Meanwhile, drug models using drug‐dependent parameters were developed. In combination with the corresponding population model, they were verified by using clinical data from Chinese adults, Caucasian adults and Caucasian elderly subjects to ensure that the drug‐dependent parameters were inputted appropriately. The verified drug models were then applied to simulate drug concentration from Chinese elderly subjects using the newly developed population model. For all the simulations, the predicted values of AUC, *C*_max_, and CL were compared with the observed values to evaluate the model prediction performance.

### 2.1 Data collection

To generate a robust Chinese geriatric population model, comprehensive physiological data in elderly subjects of Chinese ancestry (referred to as Chinese elderly subjects) were collected. Data on age distribution, height, weight, cardiac output and serum creatinine were collected from a national survey (National physical fitness and health database, 2006–2011), involving approximate 7000 Chinese elderly subjects, the majority of whom are Han ethnicity.^19^ The survey adopted a multi‐stage and stratified random sampling method to ensure the representativeness of samples. Liver weight and kidney weight were taken from a national autopsy report, which summarized the healthy organ weights of 8273 subjects.^20^

The physicochemical parameters of the model drugs were adopted from the Simcyp compound library or public domains (e.g., Scifinder, Drug Bank, Pubchem, FDA‐approved drug labels).

The clinical data were collected from literatures written in either English or Chinese. They were included for model verification and refinement when they met the following criteria: (1) PK parameters of the clinical study are available or *C*_max_, AUC or clearance (CL) can be obtained or calculated from the concentration–time curve; (2) the number of subjects in the clinical study is greater than or equal to 4; and (3) the clinical study was conducted in subjects of either Chinese or European ancestry. Also, the clinical research was excluded for our model verification when: (1) the population in the study was not stratified by young adults and elderly subjects; and (2) the clinical trials were performed using intranasal administration route, as our primary aim was the verification of the Chinese geriatric population following intravenous (IV) and oral administration.

### 2.2 Development of Chinese geriatric population model

The Chinese geriatric population model was developed based on an inbuilt Chinese adult population model in Simcyp with demographic information, cardiac output and serum creatinine recalibrated using the physiological data collected in Chinese elderly subjects aged above 65.^14^ As illustrated in Supporting Information Figure S1, the age distribution was defined based on observed data from the national survey. Height and weight were modelled against age using a polynomial equation and an exponential equation, respectively. Body surface area (BSA) was a function of both weight and height. Previously Liu and Sheng have established and verified this relationship in the Chinese geriatric population.^21^ Thus, the equation was directly adopted here. For the cardiac output, it was modelled against BSA and age using the equation previously established in Simcyp. For serum creatinine, the value was used to calculate the glomerular filtration rate (GFR) with the Cockcroft‐Gault equation as the default equation in Simcyp. Meanwhile, a GFR cap (15–400 mL/min/1.73cm^2^) was applied to fix the calculated GFR within the defined population limits. The correlation between liver or kidney volume and BSA was the same as in the Chinese adult population model. This correlation was later verified using observed data, as described below. For the other parameters, we used the default values in the inbuilt Chinese adult population model.

To evaluate if the model can adequately describe physiological characteristics in the Chinese geriatric population, the internal validation was used for height, weight, cardiac output and serum creatinine for which the 5% and 95% percentiles of these variables from simulated 4000 elderly subjects (20 trials, 200 subjects per trial) were compared to observed data. Meanwhile, external validation was conducted for liver and kidney weight. The simulated mean values based on the equation for the Chinese adult population model was compared to the observed data from Chinese elderly subjects.

### 2.3 Development and verification of drug models

Six specific drugs with different elimination pathways were used in this study based on the availability of pharmacokinetic data from Chinese elderly subjects. Simvastatin, midazolam and theophylline are eliminated mainly via hepatic clearance and exhibit high, medium and low hepatic extraction ratio, respectively. Simvastatin and midazolam are metabolized by CYP3A4, and theophylline is metabolized by CYP1A2.^22^, ^23^ In contrast, ceftazidime, vancomycin and gentamicin are eliminated predominantly via renal excretion, without active secretion.^24^ For simvastatin, midazolam and theophylline, the drug models were directly adopted from the Simcyp compound library. For ceftazidime, vancomycin and gentamicin, the drug models were developed from the literature. For these three drugs, the simple first‐order absorption model was used to describe drug absorption, and the full PBPK model was selected to describe drug distribution. The volume of distribution at steady state was predicted according to acid–base properties of each drug and then matched to clinical observations by adjusting *K*p scalar.^25^, ^26^, ^27^ Drug total clearance and renal clearance were derived from PK studies with single IV administration. The input parameters are listed in Supporting Information Table S1.

The drug models were verified by clinical PK data from Chinese adults, Caucasian adults and Caucasian elderly subjects. The simulation was conducted by mimicking the study design in the observed clinical study. If predicted AUC, *C*_max_ and CL for most studies (more than 90%) used in model verification fell within a two‐fold difference compared to the observed data, the rationality of the model was verified.

### 2.4 Simulation of the drug concentration in Chinese geriatric population

Simulations on drug concentration of Chinese elderly subjects were carried out using the verified drug model in combination with the Chinese geriatric population model. The predicted AUC, *C*_max_ and CL values were then compared with the observed clinical data to evaluate if the newly developed population model could be applied to predict drug concentration in Chinese elderly subjects.

## 3 RESULTS

### 3.1 Physiological data in the Chinese geriatric population

As shown in Table 1, the mean body height of Chinese elderly subjects aged over 75 was 162.21 ± 6.01 cm for males and 149.40 ± 6.04 cm for females, about 1% and 2% lower, respectively, compared to those aged 65–75. The mean body weight was 61.97 ± 10.58 kg for males and 53.68 ± 9.89 kg for females aged over 75, which was approximately 5% and 7% lower, respectively, when compared to those aged 65–75. Cardiac output decreased from 5.14 ± 1.40 L/min in males and 4.14 ± 1.26 L/min in females aged 65–75 to 4.77 ± 1.28 L/min in males and 3.98 ± 1.74 L/min in females aged over 75, respectively, with a decline of about 4–7%. Females and males aged 65–75 had a mean serum creatinine of 67.68 ± 19.94 μmol/L and 83.14 ± 16.22 μmol/L, respectively, whereas they increased to 77.02 ± 18.72 μmol/L and 93.48 ± 39.6 μmol/L in females and males over 75 years old. As for liver and kidney weight, females had a slightly higher liver weight (i.e., 1230.7 ± 243.1 g) when compared to males (i.e., 1225.8 ± 255.1 g), and smaller kidney weight (i.e., 350.4 ± 52.9 g) when compared to males (i.e., 269.9 ± 61.7 g). No public data on liver or kidney were available for Chinese elderly subjects older than 75.

**TABLE 1:** Summary of physiological data collected from Chinese geriatric population

Parameters	Age (65–75 years old)			Age (>75 years old)		
	Males (*n*)	Females (*n*)	Total (*n*)	Males (*n*)	Females (*n*)	Total (*n*)
Height (cm)^28^	163.83 ± 6.29 (2552)	152.13 ± 5.74 (3061)	157.45 ± 8.36 (5613)	162.24 ± 6.01 (786)	149.40 ± 6.04 (678)	156.29 ± 8.79 (1464)
Weight (kg)^28^	65.35 ± 10.50 (2573)	57.95 ± 9.70 (3065)	61.32 ± 10.73 (5638)	61.97 ± 10.58 (793)	53.68 ± 9.89 (682)	58.14 ± 11.07 (1475)
BMI (kg m^−2^)^28^	24.32 ± 3.34 (2550)	24.99 ± 3.68 (3057)	24.68 ± 3.55 (5607)	23.51 ± 3.49 (785)	23.95 ± 3.81 (676)	23.72 ± 3.65 (1461)
Cardiac output (L min^−1^)^28^	5.14 ± 1.4 (940)	4.14 ± 1.26 (849)	4.67 ± 1.43 (1789)	4.77 ± 1.28 (258)	3.98 ± 1.74 (180)	4.45 ± 1.54 (438)
Serum creatinine (μmol L^−1^)^28^	83.14 ± 16.22 (1111)	67.68 ± 20.41 (1056)	75.61 ± 19.94 (2167)	93.48 ± 39.6 (323)	72.02 ± 18.72 (225)	84.67 ± 34.35 (548)
	Male	Female	Total	Male	Female	Total
Liver weight (g)^20^	1225.8 ± 255.1	1230.7 ± 243.1	NA	NA	NA	NA
Kidney weight (g)^20^	269.9 ± 61.7	250.4 ± 52.9	NA	NA	NA	NA

NA, not applicable

Data presented in mean ± SD (*n*)

### 3.2 Development of the Chinese geriatric population model

Physiological data were then used to develop the Chinese geriatric population model in Simcyp by recalibrating the equations for describing the change of these physiological characteristics with age in the Chinese adult population model. Table 2 shows the recalibrated equations.

**TABLE 2:** The recalibrated equations used in Chinese geriatric population model

Type	Parameters	Recalibrated equations
Demographic	Age distribution	Weibull
	Age‐height relationship	*HT*_*M*_ = 166.7+0.1356 × *age* − 0.002489 × *age* ^2^
		*HT*_*F*_ = 154.6+0.1889 × *age* − 0.003178 × *age* ^2^
	Body surface area	*BSA* = 0.0151 × *WT* ^0.4259^ × *HT* ^0.5751^
Cardiac	Cardiac output	*CO* = *BSA* × 205.9 − *BSA* × *age* × 0.501
Kidney	Serum creatinine (μmol/L)	For male aged 65–75, 83.14 ± 15.89; >75, 93.48 ± 39.6, for female aged 65–75, 67.68 ± 30.16; >75, 72.02 ± 25.99
	GFR cap	15–400 mL/min/1.73cm^2^

The updated population model was used to simulate 4000 Chinese elderly subjects. In general, the 5% and 95% percentiles of simulated height, weight, cardiac output, serum creatinine as well as the mean value of liver and kidney weight were comparable to the observed data at the age over 65, suggesting that the new Chinese geriatric population model was able to describe the physiological characteristics in the Chinese geriatric population (see Table 3).

**TABLE 3:** Simulated and observed physiological parameters in Chinese geriatric population

Parameters		Age (65–75 years old)			Age (>75 years old)		
		5% percentile	95% percentile	Mean	5% percentile	95% percentile	Mean
Height (cm)^28^	Observed	144.8	171.2	157.5	142.0	170.0	156.3
	Predicted	144.4	172.7	158.6	141.1	170.3	155.6
Weight (kg)^28^	Observed	44.5	80.0	61.3	42.0	77.9	58.1
	Predicted	47.2	88.5	66.6	45.8	86.1	64.8
Cardiac output (L min^−1^)^28^	Observed	175.2	412.8	280.2	166.2	400.8	267.0
	Predicted	236.4	332.4	283.8	221.8	316.0	268.3
Serum creatinine (μmol L^−1^)^28^	Observed	50.0	105.0	75.6	53.0	125.6	84.7
	Predicted	49.6	105.8	76.1	46.5	142.6	85.1
		Male	Female		Male	Female	
^a^Liver weight (g)^20^	Observed	1225.8 ± 255.1	1230.7 ± 243.1		NA	NA	
	Predicted	1173.4 ± 190.9	1051.1 ± 178.7		NA	NA	
^a^Kidney weight (g)^20^	Observed	269.9 ± 61.7	250.4 ± 52.9		NA	NA	
	Predicted	287.4 ± 72.5	243.4 ± 62.7		NA	NA	

NA, not applicable

^a^ Liver weight and kidney weight were presented in mean ± SD.

### 3.3 Development and verification of drug models

**FIGURE 2:**
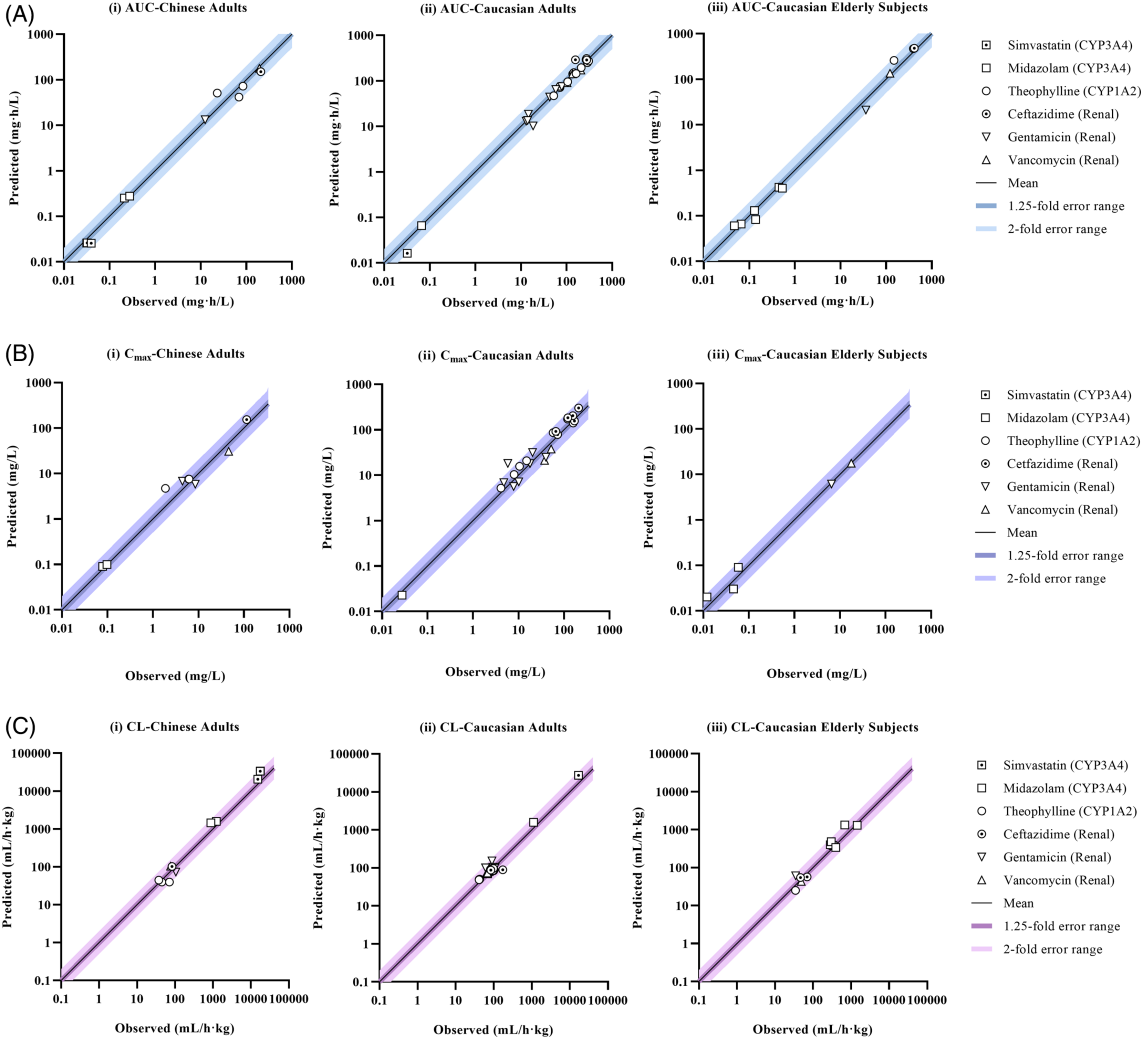
(A) Simulated area under curve (AUC) in (i) Chinese adults, (ii) Caucasian adults, and (iii) Caucasian elderly subjects. Solid black line represents the identity line. (B) Simulated maximum plasma concentration (*C*_max_) in (i) Chinese adults, (ii) Caucasian adults, and (iii) Caucasian elderly subjects. Solid black line represents the identity line. (C) Simulated clearance (CL) in (i) Chinese adults, (ii) Caucasian adults, and (iii) Caucasian elderly subjects. Solid black line represents the identity line

Drug‐dependent parameters for simvastatin, theophylline, gentamicin, vancomycin and ceftazidime were adopted from the Simcyp library or developed based on *in vitro* and clinical data. The drug models were verified using clinical data from Caucasian adults, Chinese adults and Caucasian elderly subjects (Supporting Information Table S2). Figure 2 illustrates that more than 95% predicted AUC, *C*_max_, and CL values were within two‐fold of the observed values, indicating the drug models for all six drugs were verified.

### 3.4 Verification of the Chinese geriatric population model

**FIGURE 3:**
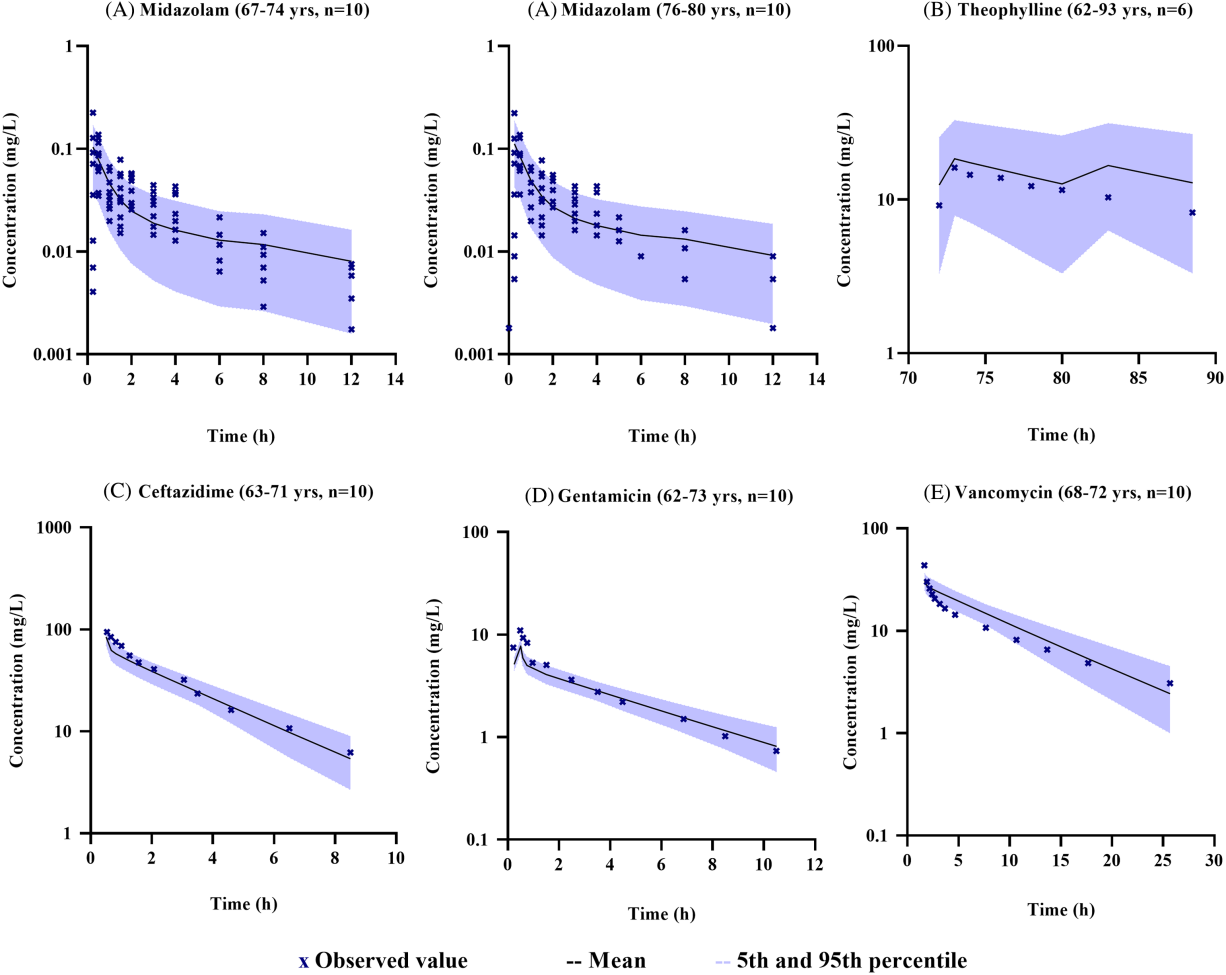
Simulated and observed plasma concentration–time profiles in Chinese geriatric population for (A) midazolam, (B) theophylline, (C) ceftazidime, (D) gentamicin and (E) vancomycin

The verified drug models were combined with the newly developed population model to predict drug concentration in Chinese elderly subjects. As illustrated in Figure 3, PBPK models could well describe the PK profiles of midazolam, theophylline, ceftazidime, vancomycin and gentamicin following the administration of drugs. For simvastatin, due to lack of concentration data reported in the literature for Chinese elderly subjects, no comparison of PK profile was possible. Besides, the predicted AUC, *C*_max_ and CL values were within the two‐fold error range of observed values for all six drugs, among which 70% prediction on AUC, 78% prediction on *C*_max_ and 70% prediction on CL were within a 1.25‐fold error range of observed values (see Table 4).

**TABLE 4:** Summary of predicted and observed PK parameters in Chinese geriatric population

	Population	Age (yrs)	Sample size	Dosing route	AUC^a^			CL^b^			C_max_ ^a^		
					Pre	Obs	Ratio	Pre	Obs	Ratio	Pre	Obs	Ratio
Simvastatin	Chinese^29^	60–91	30	20 mg, Oral	13.06	17.21	0.76	16.24	19.80	0.82	4.00	NA	NA
Midazolam	Chinese^18^	67–74	10	15 mg, Oral	346.13	228.95	1.51	1.00	1.11	0.90	107.04	97.95	1.09
		76–80	10	15 mg, Oral	394.55	254.00	1.55	0.89	0.93	0.95	116.53	96.80	1.20
Theophylline	Chinese^17^, ^30^	62–93	6	157.8 mg, Oral	128.10	103.00	1.24	24.58	36.50	0.67	19.87	16.12	1.23
		60–70	10	200 mg, IV inf	154.67	167.80	0.92	23.55	35.30	0.67	15.45	NA	NA
Ceftazidime	Chinese^31^, ^32^, ^33^, ^34^	65–75	10	1000 mg, IV Inf	238.96	244.30	0.98	62.81	58.24	1.08	85.69	101.40	0.85
		68–73	5	1000 mg, IV Inf	227.76	234.25	0.97	61.32	58.20	1.05	85.72	NA	NA
		63–71	10	1000 mg, IV Inf	214.06	264.84	0.81	65.84	60.61	1.09	84.95	101.36	0.84
		60–73	30	1000 mg, IV Inf	239.50	NA	NA	62.81	NA	NA	84.34	79.25	1.06
Gentamicin	Chinese^35^	62–73	10	120 mg, IV inf	30.09	31.00	0.97	56.47	41.56	1.36	7.70	11.52	0.67
Vancomycin	Chinese^36^	68–72	10	1000 mg, IV inf	276.31	280.30	0.99	52.38	55.50	0.94	34.13	43.55	0.78

NA, not applicable; AUC, area under the curve; CL clearance; Pre, predicted; Obs, observed; IV, intravenous; inf, infusion; Ratio was calculated by Pre/Obs.

^a^ The unit for AUC and *C*_max_ of midazolam and simvastatin are h·μg L^−1^ and μg L^−1^. For theophylline, the unit is in h·mg L^−1^ and mg L^−1^.

^b^ The unit for CL of simvastatin and midazolam are L h^−1^ kg^−1^. For theophylline, ceftazidime, gentamicin and vancomycin, the unit is in mL h^−1^ kg^−1^.

## 4 DISCUSSION

The geriatric population exhibits significant physiological changes such as a rise in gastrointestinal pH, delayed gastric emptying, decreased body muscle, increased body fats, lower GFR, reduced number of nephrons, and decreased hepatic blood flow. Alteration of these physiological changes may affect drug ADME.^37^, ^38^, ^39^, ^40^, ^41^, ^42^, ^43^, ^44^, ^45^, ^46^ In the previous study, it was shown that the PBPK model can be used to predict drug PK in Caucasian elderly subjects by incorporating these changes. However, the study of the PBPK model to predict drug PK in Chinese elderly subjects is very limited, probably due to the lack of a robust Chinese geriatric population model. In this study, we collected physiological data, such as demographic information, cardiac output, liver weight, kidney weight and serum creatinine from Chinese elderly subjects and developed a Chinese geriatric population model in Simcyp. Our study suggests that the newly established Chinese geriatric population model can be applied to predict the concentration of drugs mainly metabolized by CYP1A2, CYP3A4 or eliminated by renal clearance in Chinese elderly subjects with reasonable accuracy.

### 4.1 Comparison of physiological parameters between Chinese and Caucasian geriatric populations

In the Caucasian geriatric population, body height declines by 2% per age decade after the age of 60, and the difference between men and women is constant.^13^ Body weight increases in Caucasian subjects in their 50s and 60s and decreases after that by about 10% in each age decade.^13^ Similar trends are observed in Chinese geriatric population.

In Caucasian males and females, cardiac output decreases by 5–10% every age decade after the age of 60.^13^ A similar trend is observed in the Chinese geriatric population, with a 5% reduction in cardiac output from 65–75 to 75 years old and above.

Compared to adults aged 18–64, the liver weight of Caucasian elderly subjects (>65 years old) generally has 10–15% and 20% reduction for women and men, respectively. For kidney weight, the reduction starts with a loss of 5% at 70, 15% at 80, and later 25% at the age of 100 in both sexes.^13^ In the Chinese population, the liver weight of 60–75 years old is 9% lower than that of men aged 18–59, and 2% for women. The kidney weight of men and women both reduce by 3% at 60–75 years old when compared to those aged 18–59 years old.

There is a progressive rise in the serum creatinine level in both males and females.^47^ The serum creatinine level in elderly subjects aged 65–75 was increased by about 5% when compared to the Caucasian population aged 20–64. In elderly subjects over 75 years old, the serum creatinine level increased by 5–15%. Similarly, this increase was also observed in the Chinese geriatric population (see Table 1).

### 4.2 Development of the Chinese geriatric population model

A novel Chinese geriatric population model was developed based on the Chinese adult population model in Simcyp. We recalibrated the height, weight, cardiac output and serum creatinine, which may potentially impact drug ADME, by using the data from the Chinese geriatric population aged 65 and above. Liver or kidney weight is usually estimated based on BSA.^48^, ^49^ However, to the best of our knowledge, the information about liver or kidney weight and their corresponding BSA is still lacking in the Chinese geriatric population, resulting in difficulty to recalibrate the relationship between liver or kidney weight and BSA in Simcyp. Thus, the equation to describe this relationship in the Chinese adult population model was adopted directly, followed by the model simulation to verify whether the same equation can be applied to the Chinese geriatric population.

MPPGL is a vital parameter to convert intrinsic clearance determined *in vitro* to *in vivo* clearance. Previous reports^17^ found that the average MPPGL level was almost identical among different age groups and not associated with age (i.e., 20–45, 46–60, and 61–75 years old) in the Chinese population. Therefore, we did not correct MPPGL during the development of our geriatric population model.

The change of enzyme CYP3A and CYP1A2 with age is still controversial (see Supporting Information Table S3). On the one hand, George et al. found that microsomal 3A content declined by about 8% for every decade of life, and Tanaka et al. indicated that the clearance of CYP1A2 decreased in the elderly (>65 years old).^50^, ^51^ On the other hand, Hunt et al. and George et al. proposed that the enzyme activity of CYP3A and CYP1A2 remain unchanged as age increases, and the change of substrate clearance is attributed mainly to the changes in liver blood flow and liver volume.^44^, ^50^ Also, the enzyme abundance data in Chinese elderly subjects are still lacking. Thus, the CYP enzyme abundance in the Chinese geriatric population was assumed to be the same as in Chinese adults in our study.

Ceftazidime, gentamicin and vancomycin have not been reported as a substrate of any renal transporter. It suggests that these drugs may not be likely to have active secretion. Hence, the number of nephrons and abundance of renal transporter in our Chinese geriatric population model was kept the same as in the Chinese adult population model assuming that they have minimal impact on model prediction performance for these three drugs.

Conflicting evidence was observed for the physiological change in the gastrointestinal tract between elderly subjects and young adults. For example, gastric emptying (GE) and intestinal transit times (ITT) in elderly subjects showed both longer and shorter durations compared to young adults.^9^ Thus, in our Chinese geriatric population model, the related absorption parameters were assumed to be the same as for the Chinese adult population, which is also the assumption made in the Caucasian geriatric population model in Simcyp.

### 4.3 Verification of the Chinese geriatric population model

The verification of the Chinese geriatric population model was conducted with two approaches. Firstly, the height, weight, cardiac output, serum creatinine, liver weight and kidney weight from 4000 Chinese elderly subjects were simulated and compared with the observed data. The result suggested that the model could well describe height, weight, cardiac output and serum creatinine in the Chinese geriatric population aged from 65 to 75 and above 75. Simulated liver and kidney weight were generally comparable to observed data in the subjects aged from 65 to 74, indicating the equation for estimating liver and kidney weight in healthy Chinese volunteers could also be applied to estimate the liver and kidney weight in the older population.

Secondly, drug models with different elimination pathways were combined with the Chinese geriatric population model were used to predict drug PK in Chinese elderly subjects. The predicted AUC, *C*_max_ and CL values were all within the two‐fold error range of the observed values, further suggesting the good predictive performance of the new population model.

### 4.4 Prediction performance of the Chinese geriatric population model

In our study, the Chinese geriatric population model was developed based on the Chinese adult population model by modifying the demographic details, cardiac output and serum creatinine. To confirm that such modification is necessary, we performed the same simulation but using the Chinese adult population model and the Caucasian geriatric population model.

**FIGURE 4:**
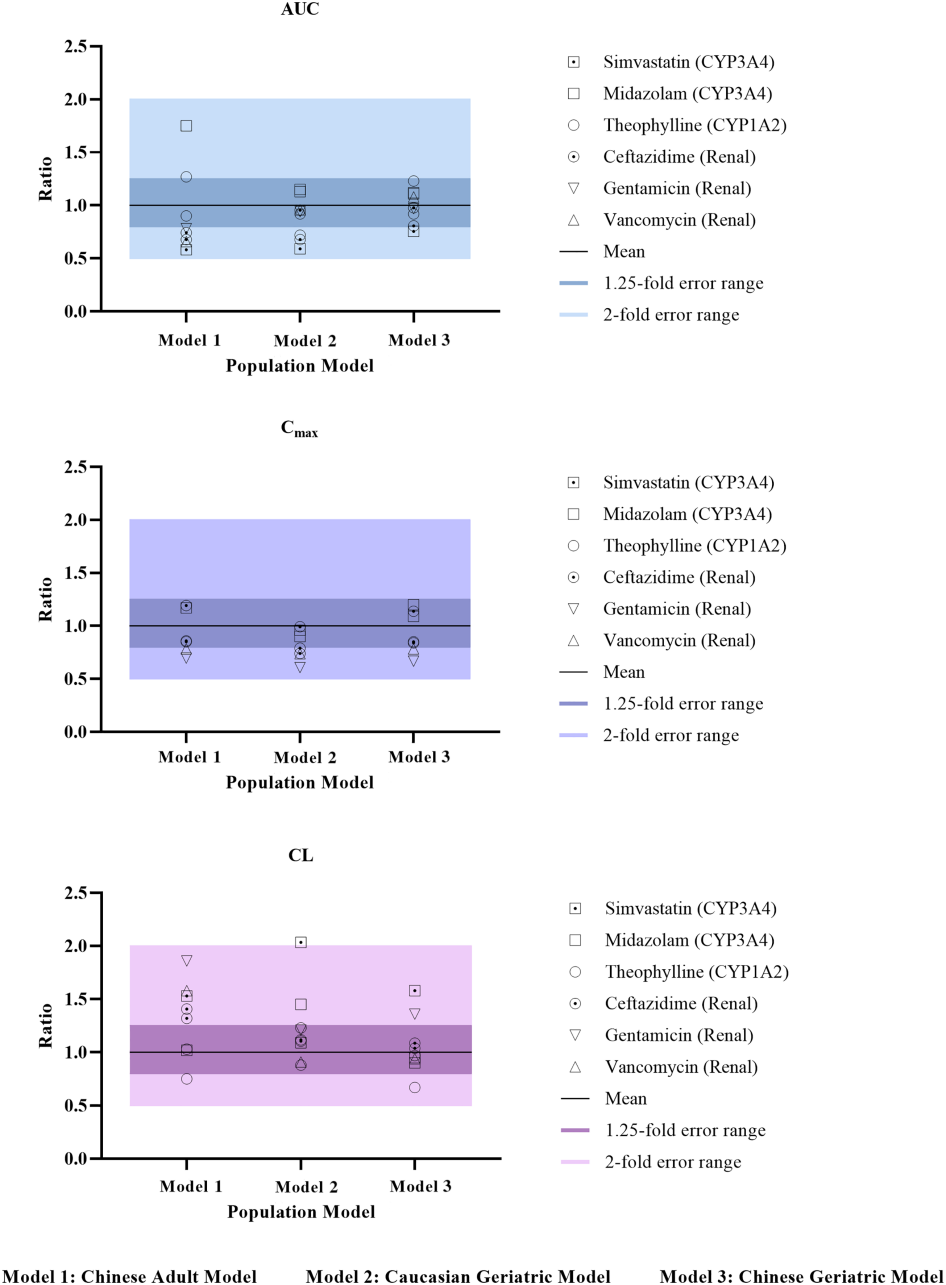
Comparison of prediction performance of different population models

As can be seen in Table S4 and Figure 4, the mean absolute prediction error (MAPE) was significantly decreased for the Chinese geriatric population model compared to the Chinese adult population model and the Caucasian geriatric population model, suggesting improved precision by our new population model. Previously Li et al. used a Chinese adult population model to predict drug exposure in the Chinese elderly subjects without changing the cardiac output and liver blood flow.^17^ It may result in poor prediction of the PK of drugs with a high or medium extraction ratio. As can be seen for simvastatin, with a high hepatic extraction ratio, the Chinese adult population model resulted in overprediction of clearance. In contrast, the Chinese geriatric population model, by incorporation of altered cardiac output, resulted in a reasonable prediction of drug clearance. Overall, the result suggested that our Chinese geriatric population model can improve the precision and accuracy of forecasts on drug PK compared to the Chinese adult population model and the Caucasian geriatric population model.

### 4.5 Limitation of the current study

In the current study, we established a Chinese geriatric population model in Simcyp. Different from the previous population model, which did not account for the change of cardiac output in the older population and thus is only capable of predicting drugs with low hepatic extraction, our population model can predict the concentration of drugs with low, medium and high hepatic extraction ratio. As there were only three drugs included in the analysis, further study will be needed to confirm the finding.

Secondly, we have confirmed that the model to estimate liver and kidney weight in healthy Chinese volunteers can also be used for older Chinese adults of 65–75 years old. However, due to a lack of data, model verification was not done in Chinese elderly subjects over 75 years old. Thus, the model should be extrapolated with caution for Chinese elderly subjects over 75 years old.

Lastly, as our new population model did not incorporate the number of nephrons and abundance of renal transporter, it may not be able to predict drugs with significant active secretion.

## 5 CONCLUSION

A Chinese geriatric population model based on more comprehensive physiological data was developed and preliminarily validated, showing that the verified drug model combined with this new population model appears to adequately predict the concentration of the drug that was metabolized by CYP1A2, CYP3A4 or eliminated by renal excretion in Chinese elderly subjects. The refinement of the ageing‐related physiological parameters such as height, weight, BSA, cardiac output and serum creatinine can significantly improve the PBPK model prediction performance on drug concentration. Such PBPK may provide the scientific rationale of the dosing regimen in the older population. Research on drugs with other elimination pathways are warranted to expand the application of this population model.

## COMPETING INTERESTS

There are no competing interests to declare.

## CONTRIBUTORS

Dongyang Liu conceived the idea and designed the study. Haiyan Li supervised the project. Cheng Cui developed the modelling and simulation strategies. Valerie Sia Jie En collected the clinical pharmacokinetic parameters and performed the modelling and simulations for drugs metabolized by CYP1A2 and CYP3A4. Xiaobei Li collected drug parameters and performed the modelling and simulations for drugs eliminated by renal excretion. Siqi Tu and Zhiheng Yu collected the physiological parameters and fitted the physiological equations to update the coefficients for Chinese elderly subjects. Valerie Sia Jie En, Zhongqi Dong and Xueting Yao contributed to data analyses, presentation or interpretation. Zhongqi Dong and Oliver Hatley revised the manuscript critically for valuable intellectual content and proofreading. Cheng Cui wrote and finalized the manuscript. Dongyang Liu gave the final approval of the version to be published.

## Supporting information

**Figure S1.** The age distribution of the Chinese geriatric population: (A) males (*n* = 4016); (B) females (*n* = 3739).
Table S1. Summary of input parameters for simvastatin, midazolam, theophylline, ceftazidime, gentamicin and vancomycin
Table S2. Verification results of drug models.
Table S3. Physiological changes and the changes in function as age increases
Table S4. Comparison of prediction performance of different population models by simulating the same clinical studies in Chinese elderly subjects

## Data Availability

The data used to support the findings of this study are available from the corresponding author upon request.
