# Supplementary material for: Development of a physiologically based pharmacokinetic (PBPK) population model for Chinese elderly subjects
Source: Br J Clin Pharmacol. 2021 May 18;87(7):2711–22. doi: 10.1111/bcp.14609 (PMC8359847; doi:10.1111/bcp.14609)
Supplement: Supplementary file 1 — Figure S1. The age distribution of the Chinese geriatric population: (A) males (n = 4016); (B) females (n = 3739). Table S1. Summary of input parameters for simvastatin, midazolam, theophylline, ceftazidime, gentamicin and vancomycin Table S2. Verification results of drug models. Table S3. Physiological changes and the changes in function as age increases Table S4. Comparison of prediction performance of different population models by simulating the same clinical studies in Chinese elderly subjects [file BCP-87-2711-s001.docx]

**Appendices**

**Table S1 CONSORT 2010 checklist of information to include when reporting a cluster randomized trial.**

| Section/Topic | Item No | Standard Checklist item | Extension for cluster designs | Page No * |
| --- | --- | --- | --- | --- |
| Title and abstract | | | |  |
|  | 1a | Identification as a randomised trial in the title | Identification as a cluster randomised trial in the title | 1 |
|  | 1b | Structured summary of trial design, methods, results, and conclusions (for specific guidance see CONSORT for abstracts) | See table 2 | 2 |
| Introduction | | | |  |
| Background and objectives | 2a | Scientific background and explanation of rationale | Rationale for using a cluster design | 4 |
|  | 2b | Specific objectives or hypotheses | Whether objectives pertain to the cluster level, the individual participant level or both | 5 |
| Methods | | | |  |
| Trial design | 3a | Description of trial design (such as parallel, factorial) including allocation ratio | Definition of cluster and description of how the design features apply to the clusters | 6 |
|  | 3b | Important changes to methods after trial commencement (such as eligibility criteria), with reasons |  |  |
| Participants | 4a | Eligibility criteria for participants | Eligibility criteria for clusters | 6 |
|  | 4b | Settings and locations where the data were collected |  | 6 |
| Interventions | 5 | The interventions for each group with sufficient details to allow replication, including how and when they were actually administered | Whether interventions pertain to the cluster level, the individual participant level or both | 7 |
| Outcomes | 6a | Completely defined pre-specified primary and secondary outcome measures, including how and when they were assessed | Whether outcome measures pertain to the cluster level, the individual participant level or both | 9 |
|  | 6b | Any changes to trial outcomes after the trial commenced, with reasons |  | NA |
| Sample size | 7a | How sample size was determined | Method of calculation, number of clusters(s) (and whether equal or unequal cluster sizes are assumed), cluster size, a coefficient of intracluster correlation (ICC or *k*), and an indication of its uncertainty | 10 |
|  | 7b | When applicable, explanation of any interim analyses and stopping guidelines |  | NA |
| Randomisation: | | | |  |
| Sequence generation | 8a | Method used to generate the random allocation sequence |  | 6 |
|  | 8b | Type of randomisation; details of any restriction (such as blocking and block size) | Details of stratification or matching if used | NA |
| Allocation concealment mechanism | 9 | Mechanism used to implement the random allocation sequence (such as sequentially numbered containers), describing any steps taken to conceal the sequence until interventions were assigned | Specification that allocation was based on clusters rather than individuals and whether allocation concealment (if any) was at the cluster level, the individual participant level or both | 6 |
| Implementation | 10 | Who generated the random allocation sequence, who enrolled participants, and who assigned participants to interventions | Replace by 10a, 10b and 10c |  |
|  | 10a |  | Who generated the random allocation sequence, who enrolled clusters, and who assigned clusters to interventions | 6 |
|  | 10b |  | Mechanism by which individual participants were included in clusters for the purposes of the trial (such as complete enumeration, random sampling) | 6 |
|  | 10c |  | From whom consent was sought (representatives of the cluster, or individual cluster members, or both), and whether consent was sought before or after randomisation | 6 |
|  |  |  |  |  |
| Blinding | 11a | If done, who was blinded after assignment to interventions (for example, participants, care providers, those assessing outcomes) and how |  | NA |
|  | 11b | If relevant, description of the similarity of interventions |  | NA |
| Statistical methods | 12a | Statistical methods used to compare groups for primary and secondary outcomes | How clustering was taken into account | 10 |
|  | 12b | Methods for additional analyses, such as subgroup analyses and adjusted analyses |  | 10 |
| Results | | | |  |
| Participant flow (a diagram is strongly recommended) | 13a | For each group, the numbers of participants who were randomly assigned, received intended treatment, and were analysed for the primary outcome | For each group, the numbers of clusters that were randomly assigned, received intended treatment, and were analysed for the primary outcome | Figure 1 |
|  | 13b | For each group, losses and exclusions after randomisation, together with reasons | For each group, losses and exclusions for both clusters and individual cluster members | Figure 1 |
| Recruitment | 14a | Dates defining the periods of recruitment and follow-up |  | 6 |
|  | 14b | Why the trial ended or was stopped |  | NA |
| Baseline data | 15 | A table showing baseline demographic and clinical characteristics for each group | Baseline characteristics for the individual and cluster levels as applicable for each group | 23 |
| Numbers analysed | 16 | For each group, number of participants (denominator) included in each analysis and whether the analysis was by original assigned groups | For each group, number of clusters included in each analysis | 12 |
| Outcomes and estimation | 17a | For each primary and secondary outcome, results for each group, and the estimated effect size and its precision (such as 95% confidence interval) | Results at the individual or cluster level as applicable and a coefficient of intracluster correlation (ICC or k) for each primary outcome | 25 |
|  | 17b | For binary outcomes, presentation of both absolute and relative effect sizes is recommended |  | NA |
| Ancillary analyses | 18 | Results of any other analyses performed, including subgroup analyses and adjusted analyses, distinguishing pre-specified from exploratory |  | Appendices |
| Harms | 19 | All important harms or unintended effects in each group (for specific guidance see CONSORT for harms) |  | NA |
| Discussion | | | |  |
| Limitations | 20 | Trial limitations, addressing sources of potential bias, imprecision, and, if relevant, multiplicity of analyses |  | 16 |
| Generalisability | 21 | Generalisability (external validity, applicability) of the trial findings | Generalisability to clusters and/or individual participants (as relevant) | 17 |
| Interpretation | 22 | Interpretation consistent with results, balancing benefits and harms, and considering other relevant evidence |  | 14 |
| Other information | | |  |  |
| Registration | 23 | Registration number and name of trial registry |  | 2 |
| Protocol | 24 | Where the full trial protocol can be accessed, if available |  | Ref no. 17 |
| Funding | 25 | Sources of funding and other support (such as supply of drugs), role of funders |  | 18 |

**Table S2 ESPACOMP Medication Adherence Reporting Guideline (EMERGE) checklist.**

| **Section** | **Item No** | **Recommendation** | **Reported on  page No / line No** |
| --- | --- | --- | --- |
| **Minimum reporting criteria** |  |  |  |
|  | 1a | **Phases of medication adherence**: State the phase(s) of medication adherence studied (i.e. initiation, implementation, and persistence) and justify, where possible, the reasons the study focuses on this/these phase(s). | 7 |
|  | 1b | **Operational definition**: Provide the precise operational/working definition for each phase of medication adherence studied (i.e., initiation, implementation, and persistence). | 7 |
|  | 1c | **Measurement**: Specify the methods of measuring medication adherence (e.g., self-report, claims data, blood sampling, electronic monitoring). Consider each phase studied (i.e., initiation, implementation, and persistence), with details on the performance of the measures (e.g., validity, reliability, and potential bias). | 7, 9, 16 |
|  | 1d | **Results**: Describe the results of the analysis appropriate to each phase of medication adherence studied (i.e., initiation, implementation, and persistence). | 12 |
|  |  |  |  |
| **Abstract** |  |  |  |
|  | 2a | Present in the abstract, in as much detail as space permits, information on the 4 minimum reporting criteria (i.e., items 1.a- 1.d). | 2 |
|  |  |  |  |

|  |  |  |  |
| --- | --- | --- | --- |
| **Background/introduction** |  |  |  |
|  | 3a | Summarize what is known about the topic with appropriate reference to the phase(s) of medication adherence (i.e., initiation, implementation, and persistence). | 4 |
|  | 3b | Describe the rationale and/or framework guiding the medication adherence study (e.g., theoretical framework and implementation science model). | 4 |
| **Study objectives or hypotheses** |  |  |  |
|  | 4a | State the study objectives or hypotheses with reference to the phase(s) of medication adherence studied and context (patient population and setting). | 5 |
|  |  |  |  |
| **Methods** |  |  |  |
| **Design & participants** | 5a | Describe the setting in which the study was done. Refer to factors relevant to medication adherence, such as characteristics of the healthcare system, organization, and the team. | 6 |
|  | 5b | State whether medication adherence was an eligibility criterion (e.g., inclusion/exclusion). If so, define the measures and rules used. | 6 |
|  | 5c | Describe routine care related to the management of medication adherence, if applicable (e.g. routine assessment of medication adherence, adherence support programs, and provider training). | 9 |
| **Measurement** | *Please refer to item 1.c. in addition to the “Measurement” item below* | |  |
|  | 6a | Measurement methods can themselves affect medication adherence (e.g., questionnaires, blood sampling, and electronic monitoring). Address this problem as appropriate. | 16 |
| **Intervention (where applicable)** | 7a | For intervention and comparator groups, describe each relevant level of the medication adherence intervention (e.g., healthcare system, organization, and provider and patient/caregiver). | 7 |
|  | 7b | Describe any implementation strategy that contributes to the translation (e.g., uptake, delivery, and sustainability) of the medication adherence intervention in clinical practice, if applicable. | NA |
| **Statistical analysis** | 8a | If medication adherence is an outcome variable, justify the statistical methods, given the characteristics of the variable (e.g., phases of medication adherence, data type, statistical distribution, data censoring, longitudinal dependence). | 10 |
|  | 8b | If medication adherence is an explanatory variable, describe how it is related to the outcome(s) (e.g., causal pathway, temporal sequence). | NA |
|  |  |  |  |
| **Results** |  |  |  |
|  | *Please refer to item 1.d in addition to the “Results” items below* | |  |
|  | 9a | Determine whether non-participation and/or dropout are associated with non-adherence, and provide any relevant data. | Figure 1 |
|  | 9b | Present sample characteristics relevant to medication adherence (e.g., those related to socio-demographics and therapy, condition, patient, caregiver, healthcare team/healthcare system). | 23 |
|  |  |  |  |
| **Discussion** |  |  |  |
|  | 10a | Discuss study strengths and limitations with reference to the phase(s) of medication adherence, where applicable (i.e., initiation, implementation, and persistence). | 16 |
|  | 10b | Discuss the study findings in the context of existing evidence on medication adherence (e.g., theory, measurement, intervention effects). | 14 |
|  | 10c | Discuss the generalizability (external validity) of the study findings with reference to the phase(s) of medication adherence, where applicable (i.e., initiation, implementation, and persistence). | 17 |

**Table S3 Organizational information of CHCs and pharmacist characteristics at each CHC.**

| **No** | **Code** | **Group** | **Community Health Centres** | | | | | **Pharmacists** | | |
| --- | --- | --- | --- | --- | --- | --- | --- | --- | --- | --- |
|  |  |  | **Medical doctors (N)** | **Pharmacists (N)** | **Nurses (N)** | **Average of diabetes patients per month (N)** | **Average of diabetes patients with hypertension per month (N)** | **Age (years)** | **Gender** | **Experience in community pharmacy (years)** |
| 1 | BBS | Intervention | 4 | 2 | 7 | 49 | 30 | 50 | Female | 2 |
| 2 | PSK | Intervention | 2 | 1 | 9 | 39 | 24 | 47 | Female | 4 |
| 3 | GRD | Intervention | 6 | 2 | 14 | 56 | 32 | 29 | Female | 4 |
| 4 | IBR | Intervention | 6 | 2 | 11 | 59 | 28 | 49 | Female | 25 |
| 5 | PTR | Intervention | 8 | 2 | 11 | 35 | 28 | 26 | Female | 4 |
| 6 | ARC | Control | 4 | 2 | 10 | 44 | 23 | 28 | Female | 10 |
| 7 | MR | Control | 5 | 2 | 8 | 48 | 20 | 49 | Male | 5 |
| 8 | NEG | Control | 4 | 1 | 5 | 30 | 20 | 26 | Female | 2 |
| 9 | CBR | Control | 2 | 2 | 6 | 47 | 22 | 27 | Female | 1 |
| 10 | PDS | Control | 7 | 2 | 11 | 35 | 16 | 26 | Female | 5 |

**Table S4. Intervention effects of the primary and secondary outcomes for the per protocol analysis with complete follow-up.**

| **Outcomes** | | **Baseline (T0)** | | **T1** | | **T2** | | **Intervention effect (T2–T0)**^a^ | | |
| --- | --- | --- | --- | --- | --- | --- | --- | --- | --- | --- |
|  |  | *Mean* ±*SD* | *N* | *Mean* ±*SD* | *N* | *Mean* ± *SD* | *N* | *Mean difference*  *(95% CI)* | *p-value* | *ICC* |
| MARS-5 sum score (5–25) | Control | 16.7 ± 3.1 | 45 | 18.4 ± 3.9 | 42 | 17.8 ± 4.6 | 45 | 4.86 (1.57; 8.16) | 0.004 | 0.386 |
|  | Intervention | 16.7 ± 2.3 | 44 | 21.8 ± 3.9 | 44 | 22.8 ± 2.9 | 44 |  |  |  |
| Systolic blood pressure (mmHg) | Control | 135.2 ± 19.5 | 45 | 133.6 ± 20.0 | 42 | 131.4 ± 16.1 | 45 | 8.05 (-8.29; 24.39) | 0.330 | -0.003 |
|  | Intervention | 132.1 ± 15.1 | 44 | 136.0 ± 17.5 | 44 | 130.2 ± 18.5 | 44 |  |  |  |
| Diastolic blood pressure (mmHg) | Control | 80.2 ± 6.2 | 45 | 78.6 ± 5.2 | 42 | 84.8 ±16.7 | 45 | -10.34 (-21.90; 1.23) | 0.079 | -0.060 |
|  | Intervention | 81.9 ± 8.0 | 44 | 80.5 ± 9.0 | 44 | 79.6 ± 9.1 | 44 |  |  |  |
| Necessity (5–25) | Control | 14.1 ± 4.2 | 45 | 14.6 ± 3.5 | 42 | 15.2 ± 3.1 | 45 | 1.97 (-0.96; 4.91) | 0.185 | -0.022 |
|  | Intervention | 14.4 ± 3.5 | 43 | 15.4 ± 3.5 | 43 | 15.9 ± 3.2 | 43 |  |  |  |
| Concern (5–25) | Control | 15.8 ± 3.4 | 45 | 15.6 ± 3.5 | 42 | 15.7 ± 3.2 | 45 | -1.59 (-4.85; 1.66) | 0.333 | -0.088 |
|  | Intervention | 15.8 ± 2.4 | 43 | 15.4 ± 3.1 | 43 | 14.6 ± 3.3 | 43 |  |  |  |
| Side effects (1–5) | Control | 2.4 ± 1.0 | 45 | 2.9 ± 1.1 | 42 | 2.7 ± 1.1 | 45 | -0.03 (-0.96; 0.90) | 0.946 | -0.059 |
|  | Intervention | 2.5 ± 1.1 | 43 | 2.5 ± 1.1 | 43 | 2.6 ± 1.3 | 43 |  |  |  |
| Necessity–concern differential (-20–20) | Control | -1.7 ± 5.2 | 45 | -1.0 ± 4.9 | 42 | -0.6 ± 4.1 | 45 | 3.56 (-0.58; 7.70) | 0.091 | 0.048 |
|  | Intervention | -1.4 ± 4.2 | 43 | 0.0 ± 4.9 | 43 | 1.3 ± 4.4 | 43 |  |  |  |

Note: ^a^: Intervention effects were adjusted for clustering effects.

Abbreviation: T1: 1-month-follow-up measurement; T2: 3-months follow-up measurement. SD: Standard deviation; CI: Confidence interval; ICC: Intraclass correlation coefficient; MARS: Medication Adherence Report Scale

**Table S5 Subgroup analysis of the intervention effects on MARS-5 based on number of concomitant drugs and diabetes complications for the per protocol analysis.**

| **Primary outcome** | **Intervention effect (T2–T0)** ^a^ | | | | | | | | | |
| --- | --- | --- | --- | --- | --- | --- | --- | --- | --- | --- |
|  | **Diabetes complications** | | | | **Number of concomitant drugs** | | | | | |
|  | With complications | | Without complications | | 1 | | 2 | | ≥ 3 | |
|  | *Mean Difference*  *(95% CI)* | *p-value* | *Mean Difference*  *(95% CI)* | *p-value* | *Mean Difference*  *(95% CI)* | *p-value* | *Mean Difference*  *(95% CI)* | *p-value* | *Mean Difference*  *(95% CI)* | *p-value* |
| MARS-5 sum score | 4.74  (0.08; 9.40) | 0.046 | 4.68  (2.85; 6.52) | <0.001 | 4.77  (1.04; 8.50) | 0.014 | 3.95  (1.43; 6.48) | 0.003 | 6.83  (3.65; 10.02) | <0.001 |

Note: ^a^: Intervention effects were adjusted for clustering effects.

Abbreviation: MARS: Medication Adherence Report Scale; CI: Confidence interval.

**Table S6 Effect of intervention: not cluster adjusted versus cluster adjusted for the intention-to-treat analysis.**

| **Outcomes** | **Effects of intervention (T2-T0)** | | | |
| --- | --- | --- | --- | --- |
|  | **Not cluster adjusted** | | **Cluster adjusted** | |
|  | Mean difference  (95% CI) | p-value | Mean difference  (95% CI) | p-value |
| MARS-5 sum score (5–25) | 4.66 (2.59; 6.73) | <0.001 | 4.62 (0.93; 8.32) | 0.008 |
|  |  |  |  |  |
| Systolic blood pressure (mmHg) | 2.03 (-6.29; 10.34) | 0.315 | 5.98 (-10.80; 22.76) | 0.241 |
|  |  |  |  |  |
| Diastolic blood pressure (mmHg) | -5.40 (-12.27; 1.48) | 0.939 | -8.61 (-20.01; 2.78) | 0.931 |
|  |  |  |  |  |
| Necessity (5–25) | -0.08 (-1.54; 1.37) | 0.545 | 1.94 (-0.78; 4.66) | 0.080 |
|  |  |  |  |  |
| Concern (5–25) | -1.17 (-2.75; 0.41) | 0.927 | -0.48 (-3.44; 2.47) | 0.627 |
|  |  |  |  |  |
| Side effects (1–5) | -0.32 (-0.81; 0.16) | 0.904 | -0.24 (-1.25; 0.78) | 0.679 |
|  |  |  |  |  |
| Necessity–concern differential (-20–20) | 1.09 (-0.82; 2.99) | 0.131 | 2.42 (-5.05; 9.89) | 0.238 |
|  |  |  |  |  |

Abbreviation: CI: Confidence interval; MARS: Medication Adherence Report Scale
